# Retention of Asymptomatic Impacted Third Molars: Effects on Alveolar Bone at the Distal Surface of Second Molars over Time

**DOI:** 10.3390/diagnostics15131643

**Published:** 2025-06-27

**Authors:** Ahmed Ata Alfurhud, Hesham Alouthah

**Affiliations:** 1 Oral and Maxillofacial Surgery and Diagnostic Sciences Department, College of Dentistry, Jouf University, King Khalid Road, Sakaka 72388, Saudi Arabia; 2 Prosthetic Dental Science Department, College of Dentistry, Jouf University, King Khalid Road, Sakaka 72388, Saudi Arabia; dr.hesham.alouthah@jodent.org

**Keywords:** alveolar bone loss, maxillofacial surgery, molar, third, periodontal diseases, periodontal pocket

## Abstract

**Objective:** To assess radiographic changes in the alveolar bone on the distal aspect of the second molars (2Ms) over time, while impacted third molars (ITMs) remain present across two timepoints. **Methods:** This retrospective observational study aimed to assess radiographic changes between two timepoints (T0 and T1). Both Orthopantomogram (OPG) and Periapical (PA) X-rays were utilized, with three measurements taken on the distal surface of 2Ms using EMAGO 6.1 software. Statistical significance was defined as a *p*-value < 0.05. **Results:** A total of 51 patients met the inclusion criteria, with a mean age of 45 years (SD ± 13). Sixty-eight second molars were assessed at baseline (T0) and follow-up (T1), with a mean interval of 20 months (SEM ± 62 days). No significant changes were found in vertical, oblique, or angular bone levels between T0 and T1. Gender significantly affected the cementoenamel junction (CEJ)–base of defect (BD) measurements (*p* = 0.022) and defect angles at T0 (*p* = 0.048), but not at the adjusted T1 (*p* = 0.292). Other variables, including medical history, smoking, and ITM angulation, showed no influence. Patient age was borderline significant in relation to intrabony defect angle (*p* = 0.047). **Conclusions:** Considering its limitations, this analysis does not provide evidence to support the hypothesis that prophylactic extraction of ITMs yields significant bone-sparing benefits. Furthermore, it does not establish that prolonged retention of ITMs consistently results in short-term bone alterations in adjacent 2Ms. Consequently, further research is warranted to more accurately assess the medium- to long-term implications of ITM retention on the bone levels of 2Ms.

## 1. Introduction

Third molar surgery (TMS) is a common procedure in the United Kingdom [1]. An international study reported impacted third molars (ITMs) in 18–68% of people [2], while a systematic review of 83,400 individuals found a prevalence of 24.40% [3]. Both non-impacted and impacted third molars can contribute to a wide spectrum of dental-related diseases, which may necessitate extraction. A retrospective study analyzed data from 1011 patients who underwent the removal of 1431 ITMs. The primary indication for extraction was pericoronitis, accounting for 49% of cases, followed by caries in 27%, distal cervical caries affecting adjacent second molars in 14%, and periodontal disease in 5% [4].

According to the guidelines issued by the United Kingdom’s National Institute for Health and Care Excellence (NICE), the removal of ITMs is indicated for various conditions (pathological association), but not for periodontal reasons related to adjacent 2Ms, as there is no reliable evidence supporting its benefits [5]. Although the Faculty of Dental Surgery (FDS) guidelines mention the possibility of removing ITMs due to periodontal disease in adjacent 2Ms, evidence to support this remains lacking [6]. A Cochrane systematic review sought evidence comparing the impact of ITM removal versus retention of pathology-free ITMs on the periodontal health of adjacent 2Ms [7]. However, the authors found a lack of high-quality evidence to support any recommendations, as the current evidence is of very low certainty and carries a significant risk of bias.

This study aimed to evaluate radiographic bone changes on the distal surface of the 2Ms at two different timepoints in proximity to ITMs to evaluate alveolar bone changes on the distal aspect of the 2Ms in the continued presence of ITMs. The hypothesis was that the retention of ITMs may affect the alveolar bone level in the distal region of the adjacent 2Ms. Additionally, the study evaluated the impact of various clinical and patient factors—such as age, gender, health status, smoking, and ITM angulation—on bone levels.

## 2. Materials and Methods

### 2.1. Study Design

This observational study, a retrospective radiographic analysis, utilized Emago^®^ radiography software.

### 2.2. Ethics

The study was conducted in accordance with the Declaration of Helsinki. Approval was obtained in advance from the Clinical Effectiveness Unit at the Royal London Dental Hospital (RLDH) (Approval ID: 13295).

### 2.3. Study Subjects

Patients who attended the Periodontal New Patient Clinics at RLDH between January 2017 and January 2019 were identified. Eligible cases included those with asymptomatic ITMs—defined as third molars free from clinical signs and symptoms—located adjacent to 2Ms and referred for periodontal treatment. The first milestone was to gather data on patients identified through records based on specific inclusion and exclusion criteria. The inclusion criteria included patients who were seen by the Periodontology Department at RLDH between January 2017 and January 2019 with ITMs near 2Ms. Additionally, these patients had to have undergone two radiographic assessments: one within the timeframe of January 2017 to January 2019 and the most recent follow-up radiograph. Patients who did not meet these criteria were excluded from the study. Patients were examined at two different timepoints: Timepoint 0 (T0), which refers to the first available full-mouth radiograph between January 2017 and January 2019, and Timepoint 1 (T1), the most recent radiograph available in the patient’s records. The findings of T0 were used as the baseline, and it then was compared to T1.

### 2.4. Radiographic Examination

**Figure 1:**
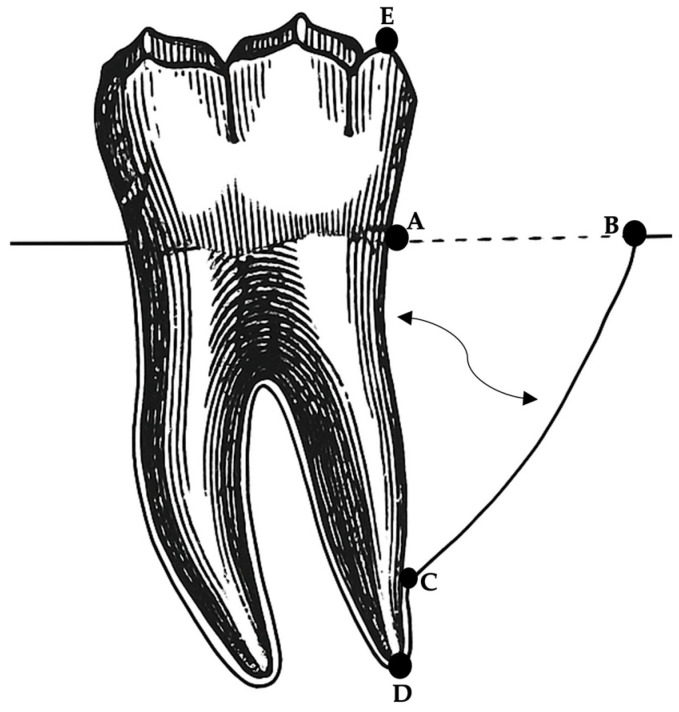
The following anatomical landmarks are described: **A** represents the CEJ, **B** represents the top of the alveolar bone crest, defined as the most coronal part of the alveolar bone, **C** represents the base of the defect, **D** represents the distal root apices of the 2Ms, and **E** represents the top of the distobuccal cusps of the 2Ms.

Radiographic examination was conducted using established anatomical landmarks and assessment criteria described by Schei et al. [8] and Björn et al. [9] for the evaluation of intrabony defects. In this study, specific reference points were identified and marked on the distal surface of each 2M, as illustrated in Figure 1, following the methodology outlined by Nibali et al. [10].

The following linear measurements were performed using Emago software: (i) a vertical line was measured in millimeters between the top of the distobuccal cusps (DC) (Point E) and the apices of the distal roots (RA) (Point D) of the 2Ms; (ii) a vertical line was measured from the identified cementoenamel junction (CEJ) (Point A) at the distal sites of the 2Ms to the deepest apical bone extension of the intrabony defect (BD) (Point C). If a restoration was present, the apical margin of the restoration was used as a fixed reference point instead of the CEJ. (iii) An oblique line was measured in millimeters from the deepest apical bone extension of the intrabony defect (BD) (Point C) to the most coronal position of the alveolar bone crest of the intrabony defect (CB) (Point B). (iv) The radiographic defect angle was determined by the two lines representing the root surface of the involved tooth: from A to C and from C to B, as previously explained by Nibali et al. [10], Steffensen et al. [11], Tonetti et al. [12], and others [13,14].

### 2.5. Radiograph Standardization

As the radiographic pairs may not be identical, the vertical distortion between the baseline radiograph (T0) and the T1 radiographs was estimated and standardized as previously described by Tonetti et al. [12] and Linares et al. [14]. To estimate this distortion, an anatomically non-variable distance (the distal crown-tooth length) from DC to RA was measured on both pairs of radiographs (from Point E to Point D), and a correction factor was calculated as follows:

- Equation one for correction factor: DC to RA (T0) ÷ DC to RA (T1) = correction factor.

To obtain the linear measurements of T1 adjusted, the following equations were used, utilizing the correction factor obtained from equation one:

- Equation two for adjusted T1 (DC to RA): DC to RA (T0) − [DC to RA (T1) × correction factor] = adjusted T1 (DC to RA)
- Equation three for adjusted T1 (CEJ to BD): CEJ to BD (T0) − [CEJ to BD (T1) × correction factor] = adjusted T1 (CEJ to BD)
- Equation four for adjusted T1 (BD to CB): BD to CB (T0) − [BD to CB (T1) × correction factor] = adjusted T1 (BD to CB).

### 2.6. Calibration of Radiographic Analysis

The measurements were carried out by a single radiographic examiner, referred to as Examiner A.A., on two separate sets of radiographs (one for T0 and another for T1), with a minimum interval of four weeks between sessions. Examiner A.A. was trained by another examiner, Examiner N.G., who served as the gold standard.

### 2.7. Data Management and Statistical Analysis

The clinical and radiographic data of patients were anonymized and entered into an Excel database, proofread for errors, and then imported into SPSS (Version 29). A descriptive analysis was conducted to evaluate various patient characteristics such as age, gender, race, smoking status, and medical and medication history. Numerical data were summarized as frequencies and percentages with standard deviation. Comparisons between timepoints (T0 and adjusted T1) were performed using the Wilcoxon signed-rank test to evaluate alveolar bone changes, with significance set at *p* < 0.05. A Mann–Whitney U test was applied to identify differences between categorical data and linear measurements, followed by the chi-square test for categorical data comparison. Finally, simple linear regression analysis was conducted to evaluate relationships between continuous variables (age) and linear measurements, with significance also set at *p* < 0.05.

## 3. Results

Data collection was carried out between June and September 2023, evaluating 1302 records from periodontal new patient clinics at RLDH for the period between January 2017 and January 2019. Of these, 1251 patients (96%) were excluded from analysis for the following reasons: a total of 948 patients had no ITMs, 196 patients had radiographs that were not taken within the required time frame, 74 patients had a single X-ray, and 33 patients lacked radiographs in their clinical records. Ultimately, 51 patients (4%) were included in the study based on the study’s inclusion and exclusion criteria.

The mean patient age was 45 years (median 44, range 22–79, SD 13). Gender distribution was 49% male and 51% female. Demographics included Caucasians (34.4%), Asians (36%), individuals of African origin (5.6%), Afro-Caribbeans (6.4%), and 17.6% with unknown ethnicity. Regarding smoking status, 77% were non-smokers, 5% were current smokers, and 18% were former smokers. Medical history showed 51% were classified as healthy (ASA I), while 49% were medically compromised (ASA II or greater). Additionally, 49% were on medication, while 51% were not. At T0, Winter’s classification showed 38.9% mesioangular, 36.7% vertical, 15.4% distoangular, 8.1% horizontal, and 0.9% inverted patterns. By T1, the distribution was 42% mesioangular, 42% vertical, 12% distoangular, 3% horizontal, and 1% inverted.

**Figure 2:**
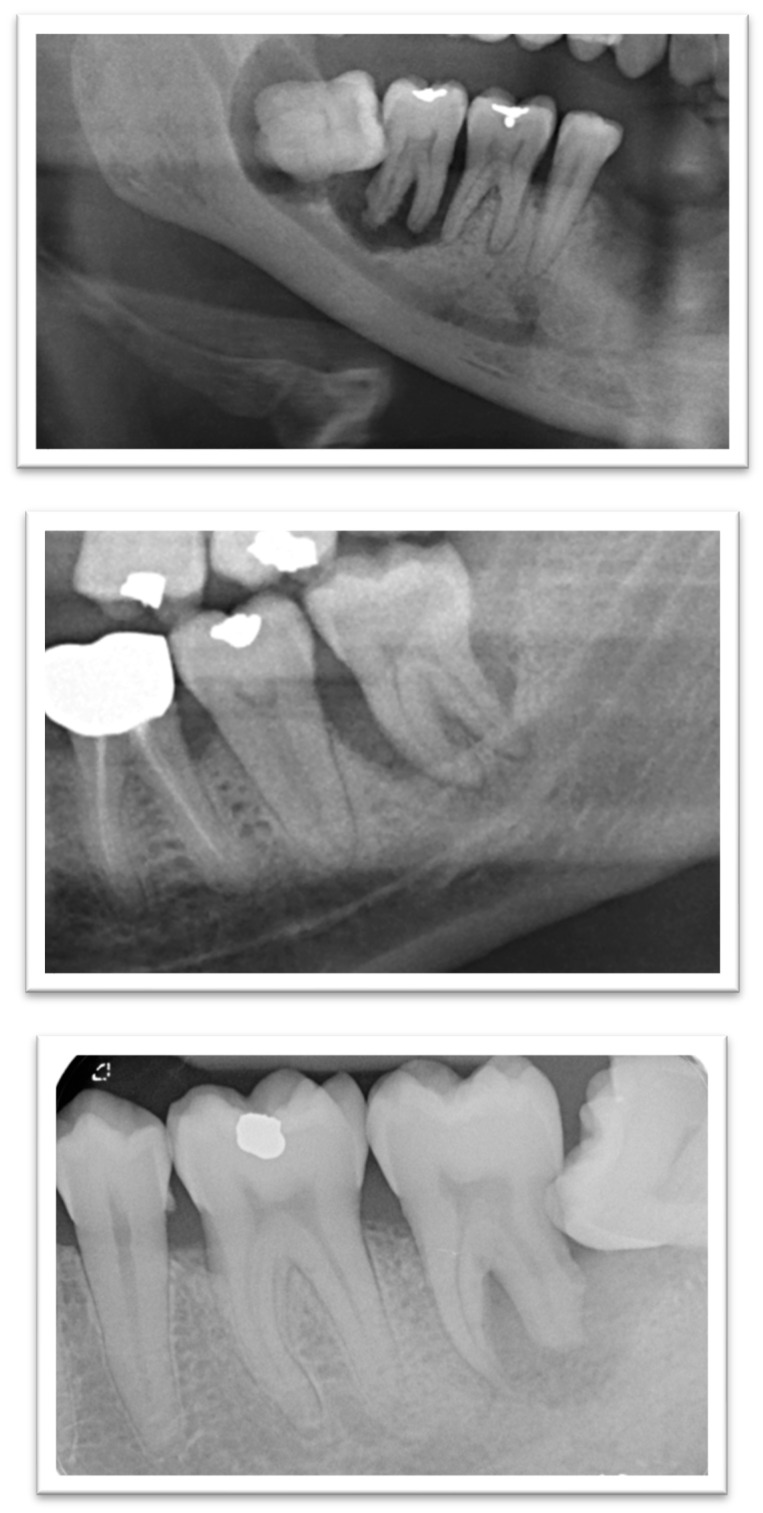
Radiographs of excluded cases with perio-endo lesions. These cases interfered with the assessment of intrabony defects due to the presence of perio-endo lesions, leading to the exclusion of three 2Ms from the final analysis.

Before statistical analysis, cases with perio-endo lesions that affected the measurement of intrabony defects were excluded, resulting in the removal of three cases, as shown in Figure 2. Two-paired radiographs were available for retrieval and analysis for 51 patients, totaling sixty-eight 2Ms at baseline (T0) and at follow-up (T1). The mean duration of the radiographic assessment period between T0 and T1 was determined to be 606 days (approximately 20 months) with a standard error of the mean estimated at 62 days.

For alveolar bone changes between T0 and adjusted T1, the Wilcoxon signed-rank test (Table 1) showed no significant difference in paired measurements. For CEJ-BD, BD-CB distances, and intrabony defect angles, *p*-values (0.377, 0.275, and 0.366, respectively) indicated the retention of the null hypothesis (*p* > 0.05).

Radiographic bone measurements were analyzed by gender at T0 and T1 using the Mann–Whitney U test. For CEJ-BD, there was a significant difference between genders at T0 (*p* = 0.022) and T1 (*p* = 0.027). For BD-CB, no significant gender difference was found at either T0 (*p* = 0.347) or T1 (*p* = 0.342). The intrabony defect angle showed a significant gender difference at T0 (*p* = 0.048), but none at T1 (*p* = 0.292), as can be shown in Table 2. No significant differences were found in the distribution of the three measurements (CEJ to BD distance, BD to CB distance, and intrabony defect angles) between healthy individuals and those with medical conditions, as well as between patients on regular medications and those not. No significant differences in alveolar bone changes were observed based on patients’ ethnicity, smoking status, or ITMs angulation. Statistical analysis confirmed this, as differences were not statistically significant at both T0 and adjusted T1, with *p*-values above 0.05, as shown in Table 2.

**Figure 3:**
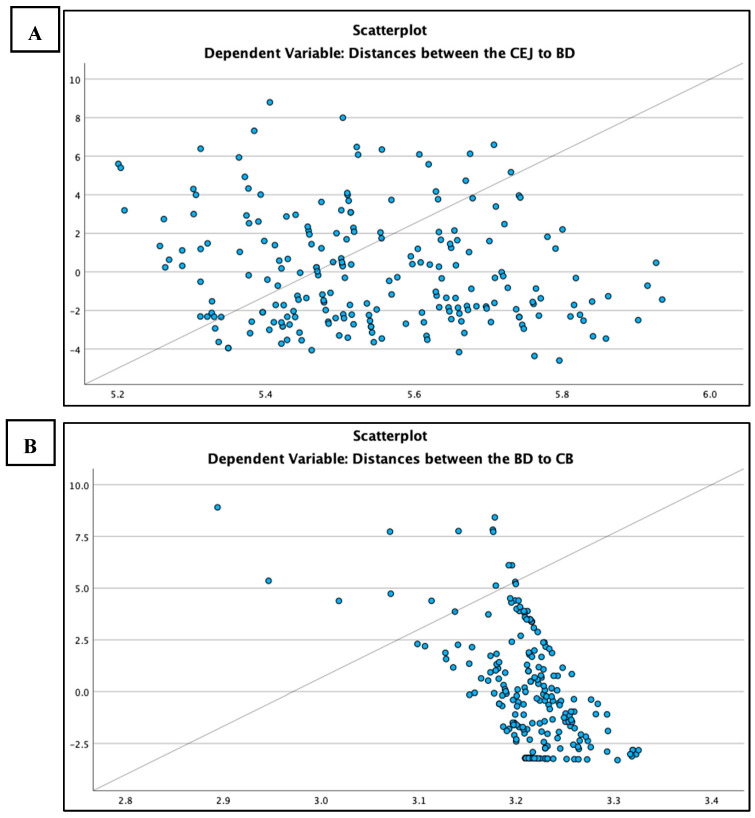

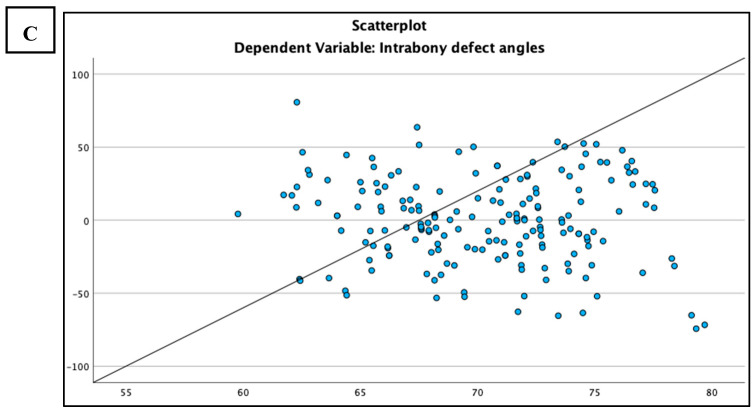
Presents scatterplots illustrating the relationship between patient age and three radiographic measurements. In (**A**), a weak and non-significant correlation is observed between patient age and the CEJ–BD distance (R = 0.056, *p* = 0.413). (**B**) shows no correlation between patient age and the BD–CB distance (R = 0.012, *p* = 0.858). In (**C**), a weak but statistically significant positive correlation is identified between patient age and the intrabony defect angle (R = 0.146, *p* = 0.047).

As part of the statistical analysis, linear regression was employed to assess the influence of patient age on the three measured parameters. As presented in Table 3, a weak correlation was observed between patient age and the distance from CEJ to the BD, with a correlation coefficient (R) of 0.056. The corresponding coefficient of determination (R^2^) was 0.003, indicating that patient age explains only 0.3% of the variability in this measurement. Furthermore, the *p*-value of 0.413 confirms that the correlation is not statistically significant, suggesting that patient age is an unreliable predictor of the CEJ–BD distance in this sample, as illustrated in Figure 3A. Similarly, no correlation was found between the distances from BD to CB and patient age, as linear regression analysis produced an R-value of 0.012 and an R^2^ of 0.000, demonstrating no linear relationship, with a *p*-value of 0.858 confirming the lack of statistical significance. These findings are depicted in Figure 3B.

In contrast, a weak positive relationship was identified between intrabony defect angles and patient age, with an R-value of 0.146 and an R^2^ of 0.021, indicating that age accounts for 2.1% of the variation in defect angles. Despite the low explanatory power, the relationship was statistically significant (*p*= 0.047), suggesting that age has a slight but detectable influence on intrabony defect angles, as illustrated in Figure 3C.

## 4. Discussion

This study aimed to evaluate changes in intrabony defects on the distal aspect of 2Ms next to ITMs over an average period of 606 days (about 20 months), from T0 to T1. The radiographic evaluation focused on short-term alveolar bone changes on the distal surface of 2Ms. The outcomes of this study suggest that the observed data does not offer sufficient evidence to establish significant differences between the two paired observations. Consequently, in the context of this study, the distances provided are not considered to be statistically different.

Although several studies in the literature have assessed bone levels on the distal surface of 2Ms, the findings of the current study are based on a unique methodology that has not previously been reported for assessing bony defects resulting from ITMs. One study assessed the relationship between retained asymptomatic third molars and periodontal pathology in adjacent 2Ms, indicating that soft tissue–ITMs significantly increased the risk of 2Ms pathology (relative risk [RR]: 4.88; 95% confidence interval [CI]: 2.62–9.08), followed by bony-ITMs (RR: 2.16) and fully erupted third molars (RR: 1.74), while the absence of ITMs posed the lowest risk [14]. However, the present study found no significant changes in bone levels over time, offering a different perspective from that report.

The study in [15] has been criticized for several methodological weaknesses, including a serious confounding bias, moderate selection bias (male-only cohort), and substantial missing data (only 416 participants analyzed out of the initial 1231) [7]. Furthermore, the timing of third molar removal in some subjects prior to follow-up further undermined the integrity of the longitudinal analysis [7]. Although a correlation between the presence of ITMs and increased 2Ms pathology was demonstrated, changes in bone levels over time were not assessed. In contrast, the present study incorporated multiple timepoints and found that bone levels in 2Ms did not significantly change over time, even when ITMs were retained.

Krausz et al. [16] used a split-mouth design to examine long-term changes in alveolar bone height between 2Ms and ITMs before and after extraction, with radiographic assessments conducted pre- and post-operation over an average of 38.14 ± 8.87 months. The study compared two groups: a test group assessed before and after ITM extraction and a control group assessed twice without ITM removal. In the control group, the ITMs were retained between the two radiographic assessments, consistent with the approach used in the present study. The findings indicated that the average bone level at Timepoint 1 was 3.224 ± 0.35 mm, while at Timepoint 2, it was 3.401 ± 0.41 mm. The *p*-value of 0.2588 indicated no statistically significant difference between the two stages in the control group [16], supporting the findings of the present study. While there exists partial similarity in the methodology between the study conducted by Krausz et al. [16] and the present study, it is essential to note a divergence in the approach to measurements. Specifically, Krausz et al. [16] focused solely on a singular vertical distance on the distal surface of the 2Ms, whereas the present study incorporated two linear measurements and one angular measurement for a more comprehensive assessment. An encouraging observation can be made when comparing the indirect findings of the control group in the study by Krausz et al. [16] with the results of the current analysis; a clear positive correlation is evident between the two sets of data. In particular, both investigations reached the significant conclusion that there were no significant changes in the bone level on the distal surface during the designated time period of the control group where the ITMs were retained between the two radiographic assessments.

Although designed with different aims, a retrospective study by Kugelberg et al. [17] highlighted significant gender differences in periodontal healing after the surgical removal of lower ITMs. They found that preoperative bone height was significantly lower in males than in females, indicating more bone loss. Similarly, the findings of the present study indicated a significant difference in distribution between males and females in the distance between CEJ and BD, but not in the BD to CB distance or intrabony defect angles. This suggests that males experience higher levels of bone loss compared to females; however, this does not necessarily mean they undergo greater changes between the two timepoints.

The current investigation found no significant association between patients’ systemic health conditions and the alveolar bone level on the distal surface of 2Ms. One possible explanation could be that the presence of ITMs primarily contributes to infrabony defects. Consequently, changes may not be observed while ITMs are still present, regardless of the patient’s health status. While medical conditions and medications can contribute to the risk of periodontal disease, they are not the only determinants. Oral hygiene practices also play crucial roles, suggesting that while compromised health can be a risk factor, it is insufficient on its own to predict the extent of alveolar bone damage.

The current research examined the correlation between changes in alveolar bone and patients’ ethnic backgrounds, finding no statistically significant association at either T0 or adjusted T1. In a retrospective study, Helmi et al. [18] assessed periodontitis prevalence by evaluating average alveolar bone loss in posterior teeth using bitewing radiographs, finding greater bone loss in Asians compared to White patients, which suggests an ethnicity-based difference. A study by Wong et al. [19] examined differences in CEJ-alveolar crest distances by ethnicity using bitewing radiographs and showed that Asian individuals had a notably greater CEJ-alveolar crest distance compared to non-Asians, highlighting a significant ethnic variation within the sample. However, the findings of the present study did not support ethnicity as a significant factor in infra-bony defects. The previous studies using patient ethnicity as an indicator in alveolar bone assessment showed varied outcomes due to differences in methodology, objectives, population groups, and age categories. None considered intrabony defects on the distal surface of 2Ms adjacent to ITMs, making this study’s findings unique by comparison.

Extensive research highlights the negative impact of smoking on periodontal health, especially alveolar bone height, as shown by studies like those by Helmi et al. [18], Bergström et al. [20,21], and Kugelberg et al. [22]. However, the findings of the present study did not observe changes in infrabony defects over time, suggesting other contributing factors. One possible explanation for this difference is that the infrabony defects observed in this study were primarily caused by ITMs, which masked smoking’s effects since the underlying cause, ITMs, remained present.

Alveolar bone changes based on ITM angulation were discussed by Matzen et al. [23], who examined the angulation of ITMs, classified as mesioangular/horizontal versus other angles. They found varied results: one observer reported no significant difference (*p* = 0.001), while three others identified significant differences (*p* < 0.001). The authors concluded that marginal bone loss in adjacent 2Ms was significantly observed for mandibular third molars positioned mesioangularly or horizontally compared to others. Similarly, Dias et al. [24] compared bone loss assessments around ITMs using OPG and Cone Beam Computed Tomography (CBCT), finding that OPG underestimated bone loss, particularly for mesioangular and horizontal ITMs (*p* < 0.05). Yesiltepe et al. [25] observed marginal bone loss in 79.4% of adjacent 2Ms on CBCT scans but found no significant association between the position of ITMs and bone loss (*p* = 0.360).

Overall, the literature shows inconsistent findings regarding whether the angulation of ITMs affects alveolar bone loss in adjacent 2Ms. The present study differs by monitoring bone level changes over time according to ITM angulation, rather than measuring the immediate effects at a single timepoint. Due to these methodological differences, the results of the present study cannot be directly compared with existing studies, underscoring the novelty and importance of the present study.

The present study also investigated the correlation between patient age and the specific measurements. Previous studies have examined the impact of age on periodontal health, revealing varying results. Kugelberg et al. [22] found significant differences in intrabony defect progression by age, with younger individuals showing improvement post-extraction, while those over 30 did not. Helmi et al. [18] identified age as a significant risk factor for alveolar bone changes, noting increased bone loss in older adults. The findings of the present study align with these trends, indicating a relationship between age and alveolar bone changes, particularly with infrabony defect angles increasing as age advances. However, due to the limited statistical power in this area, further research is still needed to clarify the direct correlation between aging and changes in alveolar bone structure in relation to ITMs.

### 4.1. Study Limitations

A key limitation of the study lies in its focus on assessing radiographic changes in the alveolar bone around the 2Ms both before and after surgical intervention of ITMs; however, none of the participants underwent extraction of ITMs during the timeframe of T1, which prevented a comparison of bone levels pre- and post-extraction. Additionally, the comparatively smaller sample size may reduce the study’s statistical power, making it challenging to detect significant differences in alveolar bone changes. Other limitations and practical challenges should be considered when implementing the proposed approach in research settings. The use of two-dimensional periapical radiographs may not fully capture the three-dimensional morphology of intrabony defects. Radiograph standardization, including consistent angulation and image quality across timepoints, can be difficult to achieve in routine practice. Moreover, the longitudinal design and requirement for paired radiographs increase time and resource demands, which may not be feasible in all settings.

### 4.2. Clinical Implications

This study aimed to provide high-quality evidence to support that the prophylactic extraction of ITMs improves the outcomes of the bone structures around the 2Ms. However, the analysis yielded insufficient evidence, as most measurements showed no statistically significant differences between the two timepoints, although a few did. Consequently, the study did not seem to indicate that prophylactic ITM extraction would offer a significant bone-sparing benefit, nor does it provide evidence that ITMs contribute to increased damage to the distal surface of 2Ms in the short term. It is proposed that a further prospective study is needed to assess bone level changes over a long time span with a large sample size and confirm whether extraction would indeed improve bone levels. The current study’s findings did not provide sufficient evidence to support the hypothesis that the presence of ITMs leads to increased bone damage over time in the short term. Therefore, management decisions regarding ITMs should align with existing recommendations such as those outlined in the FDS guidelines.

## 5. Conclusions

The study aimed to evaluate the short-term radiographic effects of ITMs on the 2Ms in patients with periodontitis. It focused on alveolar bone changes around the 2Ms without ITM removal while examining patient-related factors like age, gender, health conditions, smoking habits, and ITM angulation. Findings revealed no significant changes in bone defect size over time, indicating that ITMs did not exacerbate bone loss around 2Ms in the short term. While gender showed some influence on bone distance measurements, other factors, including age and ITM angulation, had minimal impact on bone levels. This study contributes to an ongoing debate about ITM removal in periodontal disease. While NICE guidelines recommend against routine ITM removal without active disease, FDS guidelines support removal to protect periodontal health. However, the study found no strong evidence that ITMs significantly increase bone damage near 2Ms, suggesting that prophylactic ITM removal may not be warranted for bone health alone.

## Figures and Tables

**Table 1:** Analysis of alveolar bone changes over time (T0 to T1) using Wilcoxon signed-rank test results.

Measurement	*p*-Values	Decision
CEJ to BD at T0 and adjusted T1	0.377	Retain the null hypothesis.
BD to CB at T0 and adjusted T1	0.275	Retain the null hypothesis.
Intrabony defect angle at T0 and T1	0.366	Retain the null hypothesis.

Statistical significance of *p*-value < 0.05. Abbreviations: CEJ: cementoenamel junction, BD: base of defect, CB: crestal bone, T0: Timepoint 0, T1: Timepoint 1.

**Table 2:** Significance levels of various factors affecting the measurements.

Measurement	Test	Variable	T0 Significance	T1 Significance	Decision
CEJ to BD	Mann–Whitney U Test	Gender	0.022	0.027	Significant
	Mann–Whitney U Test	Systemic Health	0.784	0.994	Not significant
	Chi-Square Test	Ethnicity	0.192	0.376	Not significant
	Chi-Square Test	Smoking Status	0.841	0.423	Not significant
	Chi-Square	ITMs Angulation	0.408	0.292	Not significant
BD to CB	Mann–Whitney U Test	Gender	0.347	0.342	Not significant
	Mann–Whitney U Test	Systemic Health	0.950	0.688	Not significant
	Chi-Square Test	Ethnicity	0.175	0.218	Not significant
	Chi-Square Test	Smoking Status	0.313	0.294	Not significant
	Chi-Square Test	ITMs Angulation	0.995	0.992	Not significant
Intrabony Defect Angle	Mann–Whitney U Test	Gender	0.048	0.292	Significant at T0 only
	Mann–Whitney U Test	Systemic Health	0.539	0.684	Not significant
	Chi-Square Test	Ethnicity	0.391	0.443	Not significant
	Chi-Square Test	Smoking Status	0.157	0.264	Not significant
	Chi-Square Test	ITMs Angulation	0.963	0.962	Not significant

Statistical significance of *p*-value < 0.05. Abbreviations: ITMs: impacted third molars, CEJ: cementoenamel junction, BD: base of defect, CB: crestal bone, T0: Timepoint 0, T1: Timepoint 1.

**Table 3:** Linear regression analysis of age correlation with the measurements.

Correlation of the Linear Measurements with Age	R-Value (R)	R-Squared (R^2^)	Sig.
CEJ to BD at T0 and adjusted T1	0.056	0.003	0.413
BD to CB at T0 and adjusted T1	0.012	0.000	0.858
Intrabony defect angle at T0 at T1	0.146	0.021	0.047

Statistical significance of *p*-value < 0.05. Abbreviations: CEJ: cementoenamel junction, BD: base of defect, CB: crestal bone, T0: Timepoint 0, T1: Timepoint 1.

## Data Availability

The data will be available on reasonable request from the corresponding author.
